# Validamycin Inhibits the Reproductive Capacity of *Spodoptera frugiperda* (Lepidoptera: Noctuidae) by Suppressing the Activity of Trehalase

**DOI:** 10.3390/insects17010105

**Published:** 2026-01-16

**Authors:** Fan Zhong, Sijing Wan, Shangrong Hu, Yuxin Ge, Ye Han, Xinyu Zhang, Min Zhou, Yan Li, Bin Tang

**Affiliations:** College of Life and Environmental Sciences, Hangzhou Normal University, Hangzhou 311121, China; zf605261118@163.com (F.Z.); wsjw9898@163.com (S.W.); 15850396510@163.com (S.H.); geyuxin107@163.com (Y.G.); 19011285720@163.com (Y.H.); zhxy0829@163.com (X.Z.); zhoumin810611@126.com (M.Z.); jlyk@hznu.edu.cn (Y.L.)

**Keywords:** insect growth regulation, insect population suppression, novel insecticidal mode of action, ovarian development, reproductive inhibition, vitellogenesis regulation

## Abstract

*Spodoptera frugiperda* (J. E. Smith, 1797), an omnivorous crop pest worldwide, reproduces prolifically. Validamycin, a competitive natural inhibitor of trehalase, is regarded as an effective and safe insecticide. Pupae were injected with a validamycin gradient (0.5–10 µg/µL) to block trehalase; enzyme activity and the Vitellogenin gene (*Vg*)/its receptor gene (*VgR*) expression (rpL10 reference) were subsequently quantified to determine the compound’s impact on *S. frugiperda* ontogeny and fecundity. Validamycin directly inhibited pupal membrane-bound trehalase, sharply lowering glycogen. Both pupal and adult mortality rose with dose, yielding marked abnormalities versus the Control Check (CK) group. At 0.5 μg/μL validamycin, eggs blackened and clumped in the lateral oviduct, blocking release; treated females produced far fewer eggs by day 4, exhibited ovarian atrophy, shorter lifespan, and low hatchability. The expression levels of *Vg* and *VgR* in the ovaries of the fall armyworm were consistent with the changes in the ovarian developmental phenotype. Validamycin significantly inhibited the activity of trehalase in *S. frugiperda*, severely hindering their normal eclosion and lowering the potential reproductive capacity of *S. frugiperda*. Simultaneously, it directly affects ovarian development and the lifespan of female moths. The results provide data to support the development of new methods for controlling *S. frugiperda*.

## Simple Summary

The fall armyworm is a highly destructive crop pest that now threatens global food security. Current control relies on chemical sprays and genetically modified crops, but the worm has become resistant to both, so safe, green alternatives are urgently needed. We tested a natural compound called validamycin that blocks the insect’s main energy sugar and upsets its reproduction. Tiny doses injected into pupae greatly reduced the number of eggs laid by female moths, shortened their lives, and caused many eggs to fail to hatch, leading to smaller future populations. Because validamycin comes from a soil microbe and is harmless to humans and wildlife, it could form the basis of a new generation of eco-friendly pesticides that help farmers use fewer chemicals while still protecting harvests and the environment.

## 1. Introduction

*Spodoptera frugiperda* (J. E. Smith, 1797), also known as the fall armyworm, is a major migratory pest globally. Owing to its widespread and gluttonous host plants, it poses a threat to global food security [1,2]. Adults fly hundreds of kilometers per night and reach 1500 km on wind, quickly infesting warm, host-rich areas where they breed and damage maize [3,4,5,6]. The female *S. frugiperda* lays up to 900–1500 eggs in its lifetime, and its strong reproductive ability is an important factor in its rapid adaptation to the environment and expansion [7,8]. At present, the main control measure for *S. frugiperda* is pesticide control. However, the widespread use of chemical insecticides threatens human health, non-target organisms, and the environment. Moreover, this pest has developed moderate or high resistance to chemical pesticides such as organophosphates, carbamate esters, and pyrethroids [9,10,11]. At present, there are also methods for planting genetically modified crops (such as *Bacillus thuringiensis* corn (Bt corn)) to control *S. frugiperda* [12], but it has recently been found that field populations across the Americas have developed high resistance to Bt corn expressing multiple proteins, such as Cry1F and Cry1Ac [13,14,15,16]. In this context, screening for green, environmentally friendly, safe, and effective biopesticides is crucial.

Reproduction sustains insect populations, and fertility underpins their control potential [17]. Restriction and weakening of the reproductive ability of pests is another approach to pest control. Carbohydrates directly affect insect reproduction. Trehalose, also known as insect blood glucose [18], is the main stored energy and stress metabolite in insects [19,20,21,22]. Research has shown that inhibition of trehalase can lead to impaired energy metabolism, abnormal growth and development, and even death in insects. The vitellogenin (Vg) and the vitellogenin receptor (VgR) play important roles in insect reproduction. Vg provides essential nutrients for embryonic development, and VgR mediates the uptake of Vg by oocytes [23]. As an important energy source, trehalose metabolism is beneficial for the development of ovarian oocytes and participates in the uptake of Vg by oocytes. Previous studies have shown that silencing *S. frugiperda* vitellogenin gene (*SfVg*) or the vitellogenin receptor gene (*SfVgR*) can hinder oocyte maturation and ovarian development, leading to a significant decrease in reproductive capacity [24]. In addition, studies have shown that trehalase inhibitors can reduce trehalase activity in the ovaries of silkworms, causing insufficient uptake of trehalose by Vg and affecting its synthesis, resulting in delayed ovarian development and decreased egg production [25,26,27]. In this study, validamycin decreased the glucose content, affected the expression levels of *SfVg* and *SfVgR*, and prevented the eggs from obtaining the energy necessary for the endocytosis mediated by VgR, which might lead to impaired egg absorption and female infertility. This indicates that trehalase not only participates in energy metabolism during insect growth and development but also in the regulation of ovarian development. Trehalase inhibition disrupts ovarian energy supply, curbing reproductive potential. Trehalase is an important candidate target for the development of new insecticides [28].

Validamycin is a competitive natural inhibitor of trehalase extracted from natural substances. The components of validamycin discovered in *Streptomyces hygroscopicus* include validamycin A to H, totaling eight compounds. It is regarded as an effective and safe insecticide [29]. As an analog of trehalose, it has shown strong inhibitory effects on trehalase activity in many pathogenic pests and has been proven to regulate the growth and development of various insects, with significant effects on teratogenicity and mortality [30,31,32]. At present, some new trehalase inhibitory compounds, such as piperine derivatives, have been shown to simultaneously affect insect development and reproduction [33,34,35]. However, little research has been conducted on the effects of validamycin on the reproductive capacities of insects. We hypothesize that by blocking trehalase, validamycin affects sugar metabolism and consequently suppresses the reproduction of *S. frugiperda*, providing new ideas and methods for pest control.

## 2. Materials and Methods

### 2.1. Experimental Insects Source

The insect used in this study was provided by the Zhejiang Academy of Agricultural Sciences (Hangzhou, China) and bred in the Key Laboratory of Animal Adaptation and Evolution, School of Life and Environmental Sciences, Hangzhou Normal University (Hangzhou, China). Both adults and larvae were cultured in an artificial climate chamber at a temperature of 26 ± 1 °C, relative humidity of 60 ± 10%, and a light cycle of 16 h/8 h (day/night). After the larvae had developed into pre-pupae, they were picked and cultured in a 6-well plate. They were observed every 12 h, which was recorded as the first day of pupation. The pupae from this period were used for subsequent experiments.

### 2.2. Preparation and Microinjection of Trehalase Inhibitors

Validamycin (C178990, Lot: 20306) was purchased from Dr. Ehrenxtorfer (Germany). According to the methods of Tang et al. [36] and Yu et al. [37], validamycin was diluted with deionized water (ddH_2_O) to concentrations of 0.5, 1, 2.5, 5, and 10 μg/μL to determine the lowest effective concentration of trehalase inhibitors.

On the first day of pupation, they were selected for microinjection with different concentrations of validamycin. The formulation (300 nL, corresponding to 0.15–3 μg validamycin per pupa) was introduced at the junction of the 5th and 6th ventral segments of the pupae, with ddH_2_O-injected pupae serving as the control (CK). Samples injected with different concentrations of effective erythromycin were collected from pupae processed for 24 h, 48 h, and 5 days for subsequent experiments.

### 2.3. Determination of Trehalase Activity

Trehalase activity was detected according to Tatun’s method [38,39]. Determination of enzyme activity requires sample pretreatment. After freezing in liquid nitrogen, the pupae were ground to a fine powder and aliquoted into EP tubes (three pupae per tube, n = 9; three biological replicates). First, the sample was homogenized using stainless steel balls, sonicated with phosphate-buffered saline (PBS; pH 7.0) for 30 min at 4 °C, 1000× *g*, and centrifuged for 20 min. We collected the supernatant, centrifuged at 20,800× *g*, 4 °C, 60 min, and assayed soluble trehalase activity and protein in the resulting supernatant; we resuspended the pellet in PBS for membrane-bound trehalase and protein assays. Then, the sample was mixed with 40 mM trehalose (Sigma Aldrich, Saint Louis, MO, USA) and PBS, incubated at 37 °C for 60 min, inactivated at 100 °C for 5 min, and removed from the mixture for 30 min in a 37 °C water bath. Trehalase activity was measured using a glucose (Go) detection kit (Sigma Aldrich, St. Louis, MO, USA), and the reaction was terminated by adding 12N H_2_SO_4_. The absorbance at 540 nm was measured using a microplate reader (Thermo Fisher Scientific, Waltham, MA, USA).

### 2.4. Determination of Carbohydrate Content

The sample treatment was performed prior to detection. After homogenization, 1 mL of PBS was added to the sample for ultrasonic crushing for 30 min, followed by centrifugation at 4 °C and 1000× *g* for 20 min. Next, 350 μL of the supernatant was ultracentrifuged at 4 °C and 20,800× *g* for 60 min. The resulting supernatant was used to detect trehalose and glycogen contents, while both the supernatant and suspension from ultracentrifugation were used to determine glucose content. In the second step, trehalose content was measured using the anthrone method [40]. To 30 μL of the sample, 1% sulfuric acid was added, and the mixture was incubated in a 90 °C water bath for 10 min and then an ice bath for 3 min. Subsequently, 30 μL of 30% KOH was added, followed by another 10 min incubation in a 90 °C water bath and 3 min in an ice bath. Then, 600 μL of developer (0.02 g anthrone + 100 mL of 80% H_2_SO_4_) was added, and the sample was incubated in a 90 °C water bath for 10 min before cooling in an ice bath. Absorbance was measured at 630 nm. In the third step, the detection methods for glycogen and glucose were similar, with the exception that 160 μL of trehalose was aliquoted and 32 μL of 0.1 U/L amylo-transglucosidase was added. The mixture was incubated in a 40 °C water bath for 4 h to convert it into glucose. The glucose content was then determined using a glucose (GO) detection kit, and the reaction was terminated by adding 12N H_2_SO_4_. The absorbance was detected at 540 nm.

### 2.5. Statistics on the Eclosion Rate

The processed pupae were divided into three groups, with 20–30 individuals in each group. From day 5 post-treatment, pupae were checked daily. The numbers of complete, incomplete, and abnormal eclosion were recorded and used to calculate the respective rates.

Method for determining incomplete eclosion: Adult insects that have emerged only partially (head, abdomen, or wings) from the pupae shell and still have some parts of their body connected to the pupae shell are considered incomplete eclosion. Incomplete eclosion rate = (N_i_/N_t_) × 100%, where N_i_ is the number of incomplete eclosion adults and N_t_ the total pupae. Post-eclosion morphological abnormalities, including but not limited to wing folding deformities, incomplete wing expansion, or impaired locomotion, are collectively defined as eclosion deformities. Abnormal eclosion rate = (N_a_/N_t_) × 100%, where N_a_ is the number of abnormal adults.

The phenotypes of the processed pupae during eclosion were documented photographically using a Canon EOS 50D (Canon Inc., Tokyo, Japan).

### 2.6. Analysis of Reproductive Capacity and Lifespan Parameters

From the first day of female eclosion until death, we observed whether the female laid eggs daily. If the female does not lay eggs until death, it is considered to have no reproductive ability, and the subsequent data analysis excludes interference from such female insects. The egg-laying behavior and survival status of reproductive female insects were observed daily and recorded until the death of the female adult. Female fecundity (eggs laid within 7 d of eclosion), oviposition timing, and longevity were recorded for three replicate groups of 10–20 pairs per treatment.

Egg masses were collected from the second to the seventh day after female eclosion to calculate the hatching rate of eggs. Ten egg masses per treatment were collected daily; we randomly chose one mass per mating pair, labeled treatment and egg count, and incubated individually. We recorded larval emergence within 7 d; unhatched eggs were deemed non-viable.

### 2.7. Anatomy and Total RNA Extraction of the Ovary

Female insects that had already mated on the 2nd, 4th, and 6th days after eclosion were selected to dissect the ovaries. The ovaries were dissected under a stereomicroscope (Leica, Wetzlar, Germany), and a Canon EOS 50D was used for photographic observation. Each treatment group should dissect at least three female insects and determine the ovarian development status according to Zhao’s ovarian development grading standards [41]. Adipose and ovarian tissues were used to detect changes in the expression of *sfVg* and *sfVgR* in *S. frugiperda.*

Five female insects were dissected from each treatment group, with each female insect serving as one biological replicate for a total of five biological replicates. Total RNA extraction was performed using the Trizol kit (Invitrogen, Carlsbad, CA, USA) according to the manufacturer’s instructions. After extraction, the mass of the total RNA was detected using 1% agarose, and the concentration and purity of the extracted RNA were measured using a NanoDrop^TM^ 2000 micro nucleic acid analyzer (Thermo Fisher Scientific, New York, NY, USA). The first strand of cDNA was synthesized using the PrimeScript^TM^ RT Reagent Kit with gDNA Eraser kit (Takara, Kusatsu, Japan) instructions.

### 2.8. Real Time Fluorescence Quantitative PCR (qRT PCR) Detection

The primer sequences are listed in Table 1. PCR reaction system 10 μL: SYBR Green Master mix 5 μL, ddH_2_O 3.2 μL, forward and reverse primers each 0.4 μL (10 μmol/L), cDNA sample 1 μL. Reaction procedure: Pre denature at 95 °C for 30 s, denaturation at 95 °C for 5 s, extension at 60 °C for 20 s, 40 cycles, and draw the dissolution curve at 60 °C. Non-specific amplification and amplification were performed on the Bio-Rad CFX96TM real-time PCR detection system (Hercules, CA, USA), with three technical replicates per sample.

### 2.9. Data Statistics

Statistical analyses were performed with IBM SPSS Statistics 20. Normality and homogeneity of variance were checked before further testing. One-way ANOVA or independent-samples *t*-tests were used to compare control and treatment groups. After one-way ANOVA, Tukey’s HSD (Honestly Significant Difference) range test—a ranking procedure for pairwise comparisons—was applied; means followed by different letters differ significantly (*p* < 0.05). For independent-samples *t*-tests, significance was denoted by asterisks (* *p* < 0.05, ** *p* < 0.01), and “ns” indicates no significant difference. All data are presented as mean ± SD. Figures were generated with GraphPad Prism (version 9.0).

## 3. Results

### 3.1. Changes in Trehalase Activity of Pupae After Injection of Validamycin at Different Concentrations

**Figure 1:**
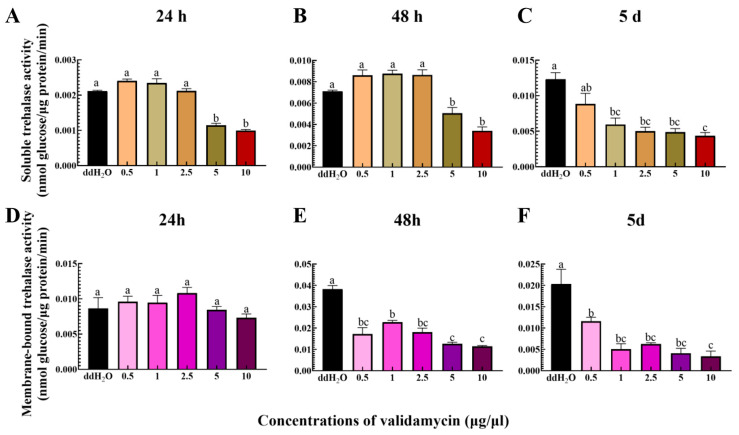
Changes in the trehalase of *S. frugiperda* pupa after 24 h, 48 h, and 5 d treatment with five different concentrations of validamycin. (**A**–**C**) Soluble trehalase, (**D**–**F**) membrane-bound trehalase. ddH_2_O: control; 0.5–10 μg/μL: validamycin concentrations. Different lowercase letters above bars indicate significant differences among treatments for the same time point (Tukey’s test, ANOVA summary: soluble 24 h, F_5,12_ = 92.52; 48 h, F_5,12_ = 29.89; 72 h, F_5,12_ = 12.67; membrane-bound 24 h, F_5,12_ = 1.69; 48 h, F_5,12_ = 35.75; 72 h, F_5,12_ = 14.82, *p* < 0.05).

The results showed that after 24 h of treatment with different concentrations of validamycin, concentrations of 5 μg/μL and 10 μg/μL significantly inhibited the soluble trehalase activity of pupae but had no significant effect on membrane-bound trehalase activity. Other concentrations of validamycin did not change the trehalase activity of *S. frugiperda* pupae 24 h after injection. After 48 h of treatment with different concentrations of validamycin (Figure 1A,D), only 5 μg/μL validamycin concentration and 10 μg/μL validamycin concentration significantly inhibited the soluble trehalase activity of pupae, while all five concentrations of validamycin significantly inhibited the membrane-bound trehalase activity (Figure 1B,E). After 5 days of treatment with different concentrations of validamycin, the soluble trehalase activity and membrane-bound trehalase activity of pupae decreased in a dose-dependent manner, with 0.5 μg/μL validamycin concentration being the lowest effective concentration for inhibition (Figure 1C,F).

### 3.2. Changes in Carbohydrate Contents of Pupae

**Figure 2:**
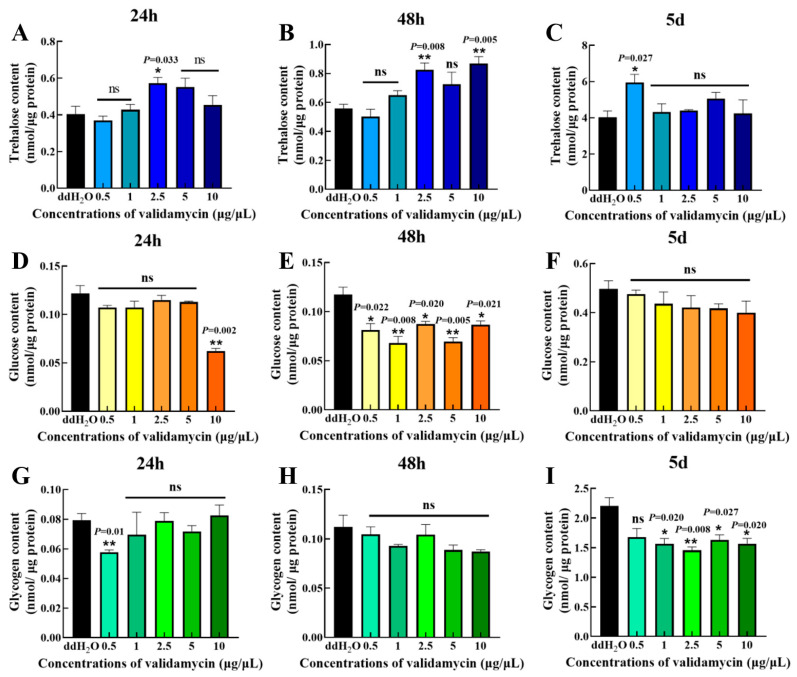
The sugar content of *S. frugiperda* pupae was affected after treatment with five different concentrations of validamycin for 24 h, 48 h, and 5 days. (**A**–**C**) Trehalose content, (**D**–**F**) glucose content, (**G**–**I**) glycogen content. Data are represented as mean ± SD. * *p* < 0.05; ** *p* < 0.01; ns, not significant, *p* > 0.05. (ANOVA summary: trehalose 24 h F_5,12_ = 4.38, 48 h F_5,12_ = 8.08, 5 d F_5,12_ = 2.59; glucose 24 h F_5,12_ = 18.53, 48 h F_5,12_ = 10.62, 5 d F_5,12_ =0.95; glycogen 24 h F_5,12_ = 1.44, 48 h F_5,12_ = 1.86, 5 d F_5,12_ = 6.12.).

Figure 2 shows that after 24 h of injection of five different concentrations of validamycin, a concentration of 2.5 μg/μL validamycin significantly increased trehalose content (Figure 2A), a concentration of 10 μg/μL validamycin significantly reduced glucose content (Figure 2D), and a concentration of 0.5 μg/μL validamycin significantly reduced glycogen content (Figure 2G). The other concentrations did not affect the changes in the three carbohydrate contents.

After injecting five different concentrations of validamycin for 48 h, compared with the CK group, the trehalose content showed an increasing trend with the increase in validamycin injection concentration. Among them, the concentrations of 2.5 μg/μL and 10 μg/μL validamycin significantly increased the trehalose content (Figure 2B). Glucose content decreased significantly at all five validamycin concentrations, whereas glycogen content remained unchanged (Figure 2E,H).

After 5 days of validamycin injection, only 0.5 μg/μL significantly increased pupal trehalose; all other concentrations had no significant effect (Figure 2C). Glucose levels remained unchanged, whereas glycogen was significantly reduced at all concentrations except 0.5 μg/μL (Figure 2F,I).

### 3.3. The Eclosion Rate, Abnormal Eclosion Rate and Adult Phenotypes

**Figure 3:**
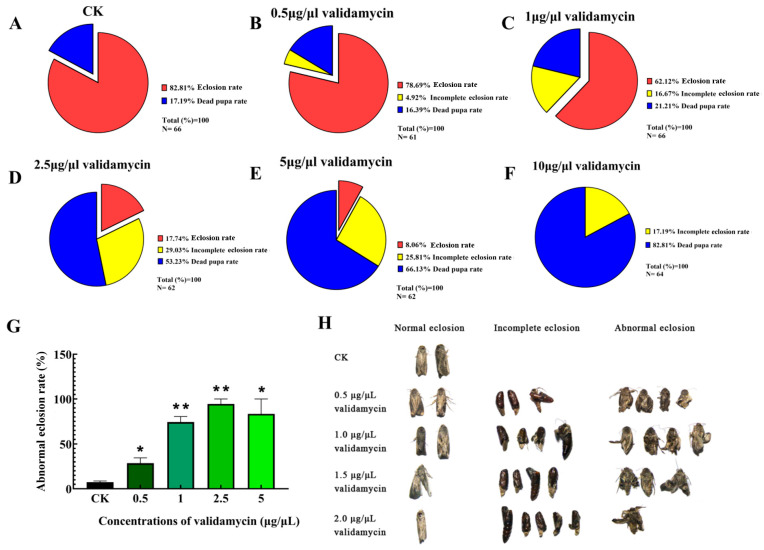
The eclosion of *S. frugiperda* was affected after five different validamycin concentrations were injected. (**A**–**F**) Eclosion rate (N represents the total number), (**G**) abnormal eclosion rate. Mean ± SD; *t*-test; * *p* < 0.05; ** *p* < 0.01, ANOVA: F_4,10_ = 18.42. (**H**) Abnormal phenotypes (no statistics were applied to this panel).

Validamycin ≥ 0.5 μg/μL induced incomplete eclosion, peaking at 29.03% under 2.5 μg/μL. At higher concentrations, defects declined while pupal mortality increased, reaching 82.81% at 10 μg/μL (Figure 3A–F). The eclosion rate declined with dose and collapsed at 10 μg/μL, leaving only incomplete eclosion or pupal death (Figure 3F–H). The statistical results of the metamorphosis rate in fully metamorphosed adults (Figure 3G) showed that the metamorphosis rate after treatment with different concentrations of validamycin was significantly higher than that in the CK group, showing an upward trend. When the injection concentration of validamycin was 1 μg/μL, the metamorphosis rate increased by 2.78 times compared with that in the CK group, and when the injection concentration was 1 μg/μL, the metamorphosis rate increased by 10 times compared with that in the CK group. With an increase in injection concentration, the eclosion rate decreased, while the eclosion abnormality rate was significantly or extremely significantly higher than the normal eclosion rate, indicating that concentrations of validamycin above 1 μg/μL can significantly inhibit the normal eclosion of *S. frugiperda*.

The abnormal phenotypes of eclosion in each treatment group indicated that the wings of the eclosion adults were wrinkled or unable to spread their wings and feet, which seriously affected their mobility. Interestingly, we observed that the abdominal phenotype of adult insects emerged normally (Figure 3H). Adults emerging after 0.5 μg/μL validamycin exhibited normal wings and flight capacity but fragile, easily fractured legs, rendering them flight-capable yet unable to climb.

Combining the inhibitory effects of five different concentrations of validamycin on the activity of trehalase in the pupae of *S. frugiperda* and the rate of abnormal eclosion, in order to ensure sufficient normal adult eclosion for subsequent experiments, a validamycin concentration of 0.5 μg/μL was used as the lowest effective concentration for subsequent experiments.

### 3.4. Changes in Ovarian Development, Egg Laying, and Hatching Rate

**Figure 4:**
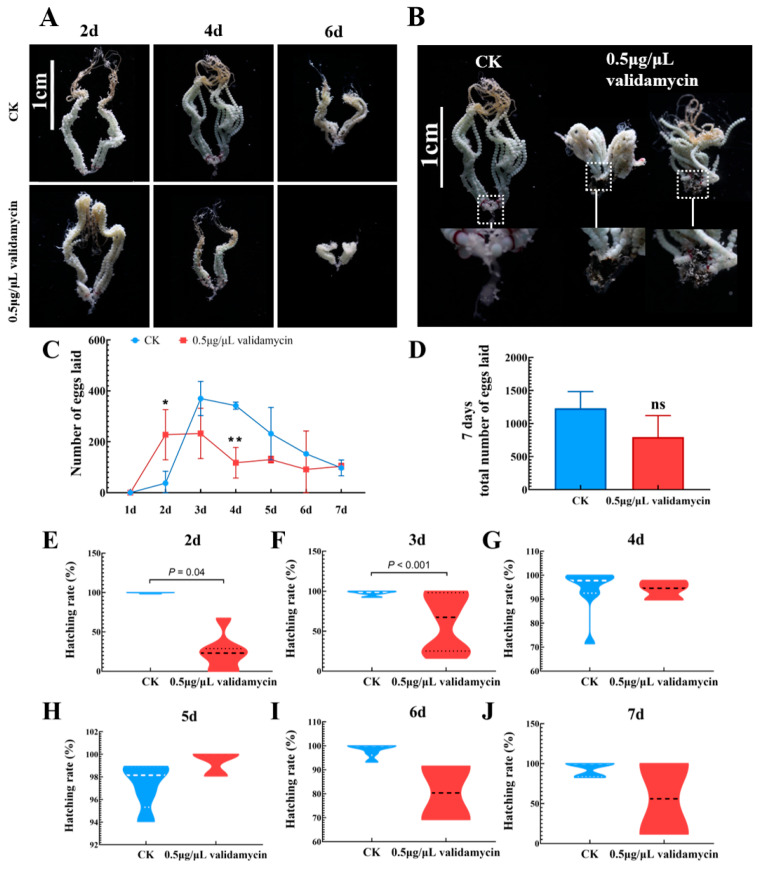
Changes in ovarian development, egg production, and egg hatchability of *S. frugiperda* within 7 days of adult life after the same pupal validamycin treatment. (**A**) The ovary of *S. frugiperda* female adult in 2 d, 4 d, and 6 d; (**B**) the infertile ovary of females; (**C**,**D**) number of eggs in single female (mean ± SD; *t*-test; * *p* < 0.05; ** *p* < 0.01; ns, not significant, *p* > 0.05; three replicate groups of 10–20 pairs per treatment); (**E**–**J**) hatchability of eggs within 7 days in females (10 egg blocks per day, with 100–200 eggs per block). The dashed and dotted lines inside indicate the median and mean hatchability, respectively.

The ovarian grades of the two groups of female *S. frugiperda* on the second day of eclosion were mainly between grades II (yolk deposition period) and III (mature waiting period), and the eggs were plump and clearly visible (Figure 4A). There was little difference in the development of ovaries on the second day of eclosion. On the fourth day of eclosion, the ovarian grades of each group were mainly distributed between grade III (mature waiting period) and grade IV (abundant production period), while the ovaries treated with 0.5 μg/μL validamycin entered grade V (late oviposition period), with significantly reduced ovarian morphology and fewer eggs than other groups. Both groups of ovaries entered the final stage of oviposition, with fewer eggs left in the 0.5 μg/μL validamycin group. This indicates that 0.5 μg/μL validamycin had a serious impact on the ovarian development of *S. frugiperda*, resulting in smaller ovaries and fewer oocytes.

Table 2 shows that all females in the CK group have reproductive ability, while 0.5 μg/μL validamycin affects the reproductive ability of females. After treatment with this inhibitor, some females showed a phenomenon of not laying eggs. Only 52.63% of females in the 0.5 μg/μL validamycin group were able to produce eggs. Further dissection of the ovaries of non-laying females in the 0.5 μg/μL validamycin group revealed that the ovarian tubes of the females were developing normally and the eggs were full. However, the eggs in the middle fallopian tubes underwent blackening and clumping, blocking the output of normal eggs (Figure 4B), which is the main reason for the female insects’ inability to reproduce.

Egg production in both groups almost reached its highest value on the third or fourth day of eclosion. The egg production of the 0.5 μg/μL validamycin group was significantly higher than that of the CK group on the second day of eclosion. Subsequently, the daily egg production was lower than that of the CK group, and on the fourth day, it was significantly lower than that of the CK group (Figure 4C). There was no significant difference in the total egg production of a single female within seven days among the groups. Although there was no significant difference in the total egg production of a single female within 7 days between the 0.5 μg/μL validamycin group and the CK group, there was a decreasing trend, manifested in a 35% reduction in egg production (Figure 4D).

The daily egg production of a single female was selected as one of the main physiological indicators to measure the reproductive capacity of female moths, and the hatching rate of laid eggs was also observed. As shown in (Figure 4E–J), the hatching rate of eggs laid by CK remained stable at over 90% in 7 days, while the median hatching rate of eggs laid by the 0.5 μg/μL validamycin group decreased to around 80%, showing a downward trend. From the hatching rate of the 0.5 μg/μL validamycin group, it can be seen that the hatching rate of eggs is higher on the 4th, 5th, and 6th days, and there are more egg blocks with lower hatching rates in the eggs laid on the 2nd, 3rd, and 7th days.

### 3.5. Changes in Pre-Oviposition, Oviposition Period, and Lifespan

After excluding the interference of non-reproductive female insects mentioned above, we observed changes in the pre-oviposition period, oviposition period, and lifespan of the two groups of female insects. As shown in Table 3, there was no significant change in the pre-oviposition period of female insects, but 0.5 μg/μL validamycin significantly shortened the oviposition period of female insects. Comparing the lifespan of the two groups of female insects, it was found that the lifespan of female insects in the CK group was longer, 13.54 days, while 0.5 μg/μL validamycin significantly shortened the lifespan of female insects, which was only half of that in the CK group.

### 3.6. Changes in sfVg and sfVgR Expression Levels in the Ovaries

**Figure 5:**
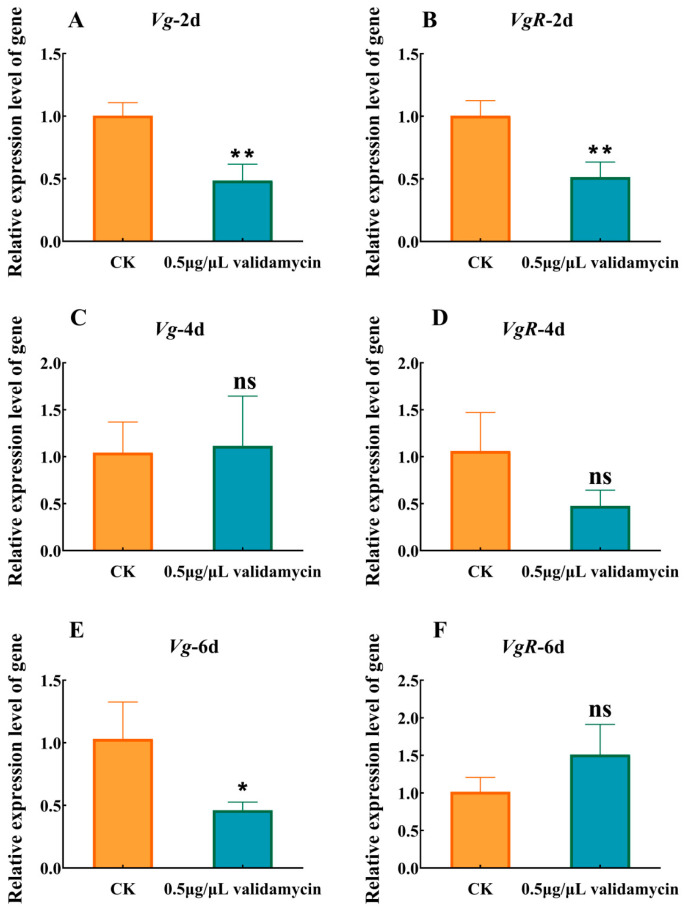
Changes in relative expression level of (**A**,**C**,**E**) *sfVg* and (**B**,**D**,**F**) *sfVgR* on female adults 2 d, 4 d, and 6 d of eclosion after validamycin treatment. CK represents control group; 0.5 μg/μL validamycin represents treatment group. Mean ± SD; Two-tailed Student’s *t*-test results: *Vg* 2 d t_7_ = 6.44, *p* < 0.001; 4 d t_4_ = 0.22, *p* = 0.83; 6 d t_4_ = 2.55, *p* = 0.06; *VgR* 2 d t_5_ = 5.35, *p* = 0.003; 4 d t_4_ = 2.24, *p* = 0.09; 6 d t_6_ = −2.43, *p* = 0.05. * *p* < 0.05; ** *p* < 0.01; ns, not significant, *p* > 0.05.

The Table shows that, compared to the CK group, 0.5 μg/μL validamycin significantly downregulated the expression levels of *sfVg* and *sfVgR* genes in the ovaries on the 2nd day of eclosion (Figure 5). On the 4th day of eclosion, *sfVg* expression levels returned to normal, while *sfVgR* expression levels remained low. On the 6th day of eclosion, 0.5 μg/μL validamycin significantly decreased *sfVg* expression levels, at which point *sfVgR* expression levels returned to normal.

## 4. Discussion

The prolific reproductive capacity of insects is a primary driver of their ecological success but simultaneously poses a significant threat to agricultural production, underscoring the importance of effective pest population control. Insect reproductive behavior is fundamental to sexual selection and evolutionary theory and offers valuable insights for pest management strategies [27,42]. Previous research has demonstrated that inhibiting trehalase activity can disrupt insect reproduction. For instance, trehalase inhibitors have been shown to reduce trehalase activity in silkworm ovaries, leading to delayed ovarian development and reduced fecundity [25]. Similarly, silencing the high trehalose hormone gene in *Blattella germanica* disrupts trehalose homeostasis in the hemolymph, significantly prolonging the oviposition period [43]. In brown planthoppers, inhibiting trehalase expression affects glucose transport and amino acid metabolism, altering ovarian nutrient allocation and energy metabolism, ultimately impacting reproductive success [44]. These findings collectively suggest a strong correlation between trehalase activity and insect reproductive capacity. In this study, we investigated the effect of micro-injected validamycin, a known trehalase inhibitor, on *S. frugiperda* pupae. Following a 48 h treatment period, validamycin significantly reduced trehalase activity in a dose-dependent manner (Figure 1). However, nominal concentrations were used throughout the study. To establish a reliable dose–response relationship, further experiments are required to measure the actual compound concentrations in the larval body fluids. Our results indicated that validamycin preferentially inhibited membrane-bound trehalase in pupae, while exhibiting a greater inhibitory effect on soluble trehalase in larvae [30]. The direct inhibition of trehalase activity by validamycin led to disturbances in carbohydrate metabolism, with glycogen concentrations significantly lower in treated pupae compared to the control group five days post-injection (Figure 2I). This reduction in glycogen is critical, as developing embryos and pupae rely on glycogen catabolism to generate ATP and the biomolecules necessary for cell proliferation and differentiation, processes essential for proper development [45]. Furthermore, we observed a dose-dependent relationship between validamycin concentration and pupal/adult mortality, with the highest mortality rate occurring at 10 μg/μL (Figure 3F). Validamycin treatment frequently resulted in the inability of pupae to complete metamorphosis into viable adults (Figure 3G). Even when metamorphosis was initiated, individuals often died before complete eclosion, resulting in 100% mortality. These findings support Tatun’s (2016) view that impaired pupal development stems from depleted glycogen reserves and insufficient energy [46].

We demonstrate that validamycin dose-dependently disrupts wing morphology in *S. frugiperda*. The eclosion rate dropped sharply relative to the control group. Higher doses intensified the defects: wings creased, hindwings failed to fold, and legs or wings remained partially extended (Figure 3). Trehalase is a key enzyme in the chitin synthesis pathway; its inhibition can lead to reduced trehalase expression, disrupting chitin metabolic balance and impacting insect molting and metamorphosis [47,48,49]. Soluble and membrane-bound trehalases perform distinct functions in insect tissues. Soluble trehalase in the hemolymph hydrolyzes intracellular trehalose, and membrane-bound trehalase is localized to the muscle [30,36,47]. Even at the lowest concentration of 0.5 μg/μL validamycin, adults flew normally but their legs often snapped. Reduced membrane-bound trehalase probably limited glucose supply to the flight muscles. Reduced membrane-bound trehalase activity limits glucose catabolism, depriving both epidermal cells and muscles of the ATP needed for post-eclosion cuticular tanning and contraction. In short, validamycin at concentrations exceeding 0.5 μg/μL demonstrably appears to affect pupal development. Considering that Lepidoptera undergo complete metamorphosis, their adult stage is dedicated to mating and reproduction. The injection of validamycin into pupae resulted in diverse developmental abnormalities (Figure 3), including wing deformities, limb loss, and reduced body size. These defects might impair female flight ability and could consequently reduce mating success. Consistent with these findings, Siriwattanarungsee et al. (2008) reported that crude extracts containing azadirachtin inhibited eclosion and reproduction rates in *Musca domestica* [50]. Similarly, Li et al. (2024) found that a mixture of tea saponin and matrine inhibited the reproductive ability of *S. frugiperda*, although the precise mechanism remains unclear [51]. Interestingly, this study observed blackening and clumping of eggs within the median oviducts of the 0.5 μg/μL validamycin group, obstructing normal egg deposition (Figure 4B). While such phenomena have not been previously reported in this context, chitin is known to be present in insect ovaries, oocytes, embryonic cuticles, and eggs [52,53]. Notably, novaluron, a chitin synthesis inhibitor, induced altered ultrastructure in the ovaries of 1-day-old *Lygus lineolaris* after 48 h of treatment, distorting follicular epithelial cell development in oocytes [54]. Silencing of *LsCHS2* in *Lepeophtheirus salmonis* resulted in oocytes with normal morphology but significantly shortened egg strings compared to controls [55]. Although cumulative egg output over 7 days did not differ, the 0.5 μg/μL group laid significantly fewer eggs than the control group on day 4 (Figure 4C). Combined with observations of ovarian atrophy, a significantly shortened lifespan, and a low egg hatching rate (Table 2, Figure 4), it is evident that 0.5 μg/μL validamycin severely compromises the reproductive capacity of females. Inhibition of chitin synthesis altered egg morphology. Given that validamycin inhibits trehalase and the chitin synthesis pathway is potentially dependent on trehalose metabolism, we hypothesized that the ovarian chitin might be affected. However, this study did not directly measure the chitin content or the expression of *CHS*/*CHT* genes. Therefore, this hypothesis still needs to be verified. In line with this, Santos et al. detected membrane-bound trehalase in the ovaries of *Rhodnius prolixus* [24,56], and Shimada & Yamashita [57] and Yu et al. [58] showed that trehalose hydrolysis is required to supply sugars to silkworm oocytes, underscoring the enzyme’s role in ovarian and egg development. Accordingly, Adhav et al. recorded a 20% drop in oviposition when insects ingested 50 ppm validamycin [59]. Dietary validamycin A lowered fecundity in *Helicoverpa armigera* by inhibiting trehalase activity in vitro and suppressing *HaTre-1* expression. Reduced *TRE1* expression, in turn, disrupts vitellogenin production and ovarian development [60]. This study’s detection of significantly reduced membrane-bound trehalase activity in pupae treated with 0.5 μg/μL validamycin (Figure 1) suggests that the decrease in reproductive capacity in this group is likely related to trehalase involvement in sugar transport during *S. frugiperda* oogenesis. Vitellogenin (Vg) and its receptor (VgR) drive oogenesis, oviposition, and embryogenesis in insects [61]. Because Vg is secreted into the hemolymph and taken up by developing oocytes via energy-dependent, receptor-mediated endocytosis, its titer is the most reliable index of female fertility [62,63]. Trehalose is the primary circulating carbohydrate used by growing oocytes after trehalase-mediated hydrolysis [64,65]. Inhibition of trehalase activity in *Periplaneta americana* can impede the energy supply required for Vg absorption by oocytes, thereby reducing Vg accumulation in the ovary [66]. Prior studies have shown that certain piperine derivatives can reduce *S. frugiperda* reproductive capacity, accompanied by decreased expression levels of *sfVg* and *sfVgR* genes [34,35,67]. This aligns with the results observed with 0.5 μg/μL validamycin (Figure 5). Consequently, we hypothesize that trehalase inhibitors may suppress trehalase activity within eggs or limit their available energy, thereby reducing egg hatching rates and suppressing *S. frugiperda* reproduction.

## 5. Conclusions

In this experiment, different concentrations of validamycin were injected into the first-day pupae of *S. frugiperda*, and the activity of trehalase in pupae at different developmental stages was measured. Macroscopic physiological indicators, such as egg production, ovarian development, and egg hatching rate of female moths after treatment, were observed. Combined with the detection of changes in the relative gene expression levels of *sfVg* and *sfVgR*, the inhibitory effect of validamycin as a trehalase inhibitor on the reproductive capacity of *S. frugiperda* was further explored. The absence of a needle-puncture-only control prevents us from separating injection trauma from compound-specific effects, and this limitation should be addressed in follow-up work.

This research has shown that Validamycin at 0.5 μg/μL curbs female *S. frugiperda* fecundity, but the mechanism remains unknown. We therefore tracked expression of reproduction-related genes and levels of energy metabolites to identify how the inhibitor reduces fertility. This will assist in screening for efficient and stable trehalase inhibitors and accelerate the development of efficient biopesticides. This study employed microinjection as the sole exposure route. Microinjection bypasses the cuticle and may overestimate actual toxicity. Consequently, the observed dose–response relationships and their translatability to field settings remain to be validated through alternative delivery modes such as foliar spraying, bait formulations, or oral ingestion in order to establish effective doses under realistic application conditions.

## Figures and Tables

**Table 1:** The primers for qRT-PCR.

Gene Name	Forward Primer (5′-3′)	Reverse Primer (5′-3′)	Gene ID
*Vg*	CGAAGAACCTCAAATACGAAACTGT	TGGTGCTGGAGTGGGTAGATAA	MT505383
*VgR*	CGACGAGTGCACTGAAGATG	GAGGCGTCAGTATCGGTGTA	XM_035595777.1
*RPL10*	GACTTGGGTAAGAAGAAG	GATGACATGGAATGGATG	

**Table 2:** Probability of fertile females after validamycin injected. Different lowercase letters indicate significant differences at the level of *p* < 0.05 (*t*-test).

	Probability	Chi-Square Test
CK	100%	a
0.5 μg/μL validamycin	52.63%	b (*p* < 0.001)

**Table 3:** The pre-oviposition period, spawning time, and longevity in female adults after pupa were injected. Different lowercase letters indicate significant differences at the level of *p* < 0.05 (*t*-test).

Unit: Days	Pre-Oviposition Period	Spawning Time	Longevity
CK	1.91 ± 0.08 a	6.67 ± 1.15 a	13.54 ± 1.05 a
0.5 μg/μL validamycin	1.70 ± 0.30 a	3.56 ± 0.51 b	6.78 ± 0.19 d

## Data Availability

The original contributions presented in this study are included in the article. Further inquiries can be directed to the corresponding authors.

## References

[1] Adnan S.M., Cattermole H., Saligari K., Spafford H. (2024). Pastoral grasses and legumes as potential host plants for fall armyworm *Spodoptera frugiperda* (J.E. Smith) development. Int. J. Trop. Insect Sci..

[2] Martinelli S., Barata R.M., Zucchi M.I., Silva-Filho M.C., Foster J.E., Omoto C. (2006). Molecular variability of *Spodoptera frugipeerda* (Lepidoptera:Noctuidae) population associated to maize and cotton crops in Brazil. J. Econ. Entomol..

[3] Johnson S.J. (1987). Migration and life history strategy of the fall armyworm, *Spodoptera frugiperda*, in the Western Hemisphere. Insect Sci. Applic..

[4] Goergen G., Kumar P.L., Sankung S.B., Togola A., Tamò M. (2016). First report of outbreaks of the fall armyworm *Spodoptera frugiperda* (J. E. Smith) (Lepidoptera: Noctuidae), a new alien invasive pest in West and Central Africa. PLoS ONE.

[5] Midega C.A.O., Pittchar J.O., Pickett J.A., Hailu G.W., Khan Z.R. (2018). A climate-adapted push-pull system effectively controls *Spodoptera frugiperda* (Lepidoptera: Noctuidae) in maize in East Africa. Crop Prot..

[6] Montezano D.G., Sosa-Gómez D.R., Roque-Specht V.F., Sousa-Silva J.C., Paula-Moraes S.V., Peterson J.A., Hunt T.E. (2018). Host plants of *Spodoptera frugiperda* (Lepidoptera: Noctuidae) in the Americas. Afr. Entomol..

[7] Sparks A.N. (1979). A Review of the biology of the fall armyworm. Florida Entomol..

[8] Kenis M., Benelli G., Biondi A., Paul-André C., Roger D., Nicolas D., Rhett D.H., Darren K., Ivan R., van den Johnnie B. (2023). Invasiveness, biology, ecology, and management of the fall armyworm, *Spodoptera frugiperda*. Entomol. Gen..

[9] Desneux N., Decourtye A., Delpuech J.M. (2007). The sublethal effects of pesticides on beneficial arthropods. Annu. Rev. Entomol..

[10] Zheng H., Xie W., Fu B., Xiao S., Tan X., Ji Y., Cheng J., Wang R., Liu B., Yang X. (2012). Annual analysis of field-evolved insecticide resistance in *Bemisia tabaci* across China. Pest Manag. Sci..

[11] Khan T., Khan H.A.A., Haider M.S., Anwar W., Akhter A. (2023). Selection for resistance to pirimiphos-methyl, permethrin and spinosad in a field strain of *Sitophilus oryzae*: Resistance risk assessment, cross-resistance potential and synergism of insecticides. Environ. Sci. Pollut. Res. Int..

[12] Roger D., Phil A., Melanie B., Tim B., Victor C., Matthew C., Yelitza C., Natalia C., Regan E., Julien G. (2017). Fall armyworm: Impacts and implications for Africa. Outlooks Pest Manag..

[13] Storer N.P., Babcock J.M., Schlenz M., Meade T., Thompson G.D., Bing J.W., Huckaba R.M. (2010). Discovery and characterization of fieldresistance to Bt maize: *Spodoptera frugiperda* (Lepidoptera: Noc-tuidae) in Puerto Rico. J. Econ. Entomol..

[14] Jakka S.R., Knight V.R., Jurat-Fuentes J.L. (2014). Fitness costs associat-ed with field-evolved resistance to Bt maize in *Spodoptera frugiperda* (Lepidoptera: Noctuidae). J. Econ. Entomol..

[15] Banerjee R., Hasler J., Meagher R., Banerjee R., Hasler J., Meagher R., Nagoshi R., Hietala L., Huang F., Narva K. (2017). Mechanism and DNA-based detection of field-evolved resistance to transgenic Bt corn in fall armyworm (*Spodoptera frugiperda*). Sci. Rep..

[16] Stirle J.L., Matias J.E.F., Mendes G.R., Moscardini V.F., Maia J.B., Michaud J.P., Gontijo P.C. (2024). Differential susceptibility of *Spodoptera frugiperda* (Lepidoptera: Noctuidae) to single versus pyramided Bt traits in Brazilian soybean: What doesn’t kill you makes you stronger?. Pest Manag. Sci..

[17] Roy S., Saha T.T., Zou Z., Raikhel A.S. (2018). Regulatory pathways controlling female insect reproduction. Annu. Rev. Entomol..

[18] Shukla E., Thorat L.J., Nath B.B., Gaikwad S.M. (2015). Insect trehalase: Physiological significance and potential applications. Glycobiology.

[19] Wegener G., Tschiedel V., Schloder P., Ando O. (2003). The toxic and lethal effects of the trehalase inhibitor trehazolin in locusts are caused by hypoglycaemia. J. Exp. Biol..

[20] Merzendorfer H., Zimoch L. (2003). Chitin metabolism in insects: Structure, function and regulation of chitin synthases and chitinases. J. Exp. Biol..

[21] Wegener G., Macho C., Schlöder P., Kamp G., Ando O. (2010). Long-term effects of the trehalase inhibitor trehazolin on trehalase activity in locust flight muscle. J. Exp. Biol..

[22] Chen J., Zhang D., Yao Q., Zhang J., Dong X., Tian H., Chen J., Zhang W. (2010). Feeding-based RNA interference of a trehalose phosphate synthase gene in the brown planthopper, *Nilaparvata lugens*. Insect Mol. Biol..

[23] Han S., Wang D., Song P., Zhang S., He Y. (2022). Molecular characterization of vitellogenin and its receptor in *Spodoptera frugiperda* (J. E. Smith, 1797), and their function in reproduction of female. Int. J. Mol. Sci..

[24] Santos R., Mariano A.C., Rosas-Oliveira R., Pascarelli B., Machado E., Meyer-Fernandes J., Gondim K. (2008). Carbohydrate accumulation and utilization by oocytes of *Rhodnius prolixus*. Arch. Insect Biochem. Physiol..

[25] Katagiri N., Ando O., Yamashita O. (1998). Reduction of glycogen in eggs of the silkworm, *Bombyx mori*, by use of a trehalase inhibitor, trehazolin, and diapause induction in glycogen-reduced eggs. J. Insect Physiol..

[26] Lu K., Wang Y., Chen X., Zhang X., Li W., Cheng Y., Li Y., Zhou J., You K., Song Y. (2018). Adipokinetic hormone receptor mediates trehalose homeostasis to promote vitellogenin uptake by oocytes in *Nilaparvata lugens*. Front. Physiol..

[27] Anholt R.R.H., O’Grady P., Wolfner M.F., Harbison S.T. (2020). Evolution of Reproductive Behavior. Genetics.

[28] Matassini C., Parmeggiani C., Cardona F. (2020). New frontiers on human safe insecticides and fungicides: An opinion on trehalase inhibitors. Molecules.

[29] Iwasa T., Higashide E., Yamamoto H., Shibata M. (1971). Studies on validamycins, new antibiotics. II. Production and biological properties of validamycins A and B. J. Anti..

[30] Luo Y.J., Chen Y., Wang X.J., Wang S.T., Yang Y.Y., Xu H.X., Qu C., Wu Y., Li C., Wang S.G. (2022). Validamycin affects the chitin metabolism and development in fall armyworm (*Spodoptera frugiperda* J.E. Smith) by inhibiting trehalase and chitinase activities. Entomol. Gen..

[31] Li Y., Xu Y., Wu S., Wang B., Li Y., Liu Y., Wang J. (2023). Validamycin Inhibits the Synthesis and Metabolism of Trehalose and Chitin in the Oriental Fruit Fly, *Bactrocera dorsalis* (Hendel). Insects.

[32] Zhong F., Jiang X., Chao L., Chen Y., Wang S., Chao L., Jiang Z., He B., Xu C., Wang S. (2023). Potential inhibitory effects of compounds ZK-PI-5 and ZK-PI-9 on trehalose and chitin metabolism in *Spodoptera frugiperda* (J.E. Smith). Front. Phys..

[33] Yu L.H., Zhong F., Jiang X.Y., He B., Fu H., Liu X., Mao Q., Zhao Y., Wang S., Wu Y. (2023). Effect of Three Novel Thiazolidiones on the Development, Reproduction, and Trehalase Activity of *Spodoptera frugiperda* (Lepidoptera: Noctuidae). Agronomy.

[34] Tang B., Hu S.R., Luo Y.J., Shi D.M., Liu X.Y., Zhong F., Jiang X.Y., Hu G., Li C., Duan H.X. (2024). Impact of Three Thiazolidinone Compounds with Piperine Skeletons on Trehalase Activity and Development of *Spodoptera frugiperda* Larvae. J. Agr. Food Chem..

[35] Tang B., Han Y., Mao Q.X., Fu H.Y., Luo Y.J., Hua L.Y.H., Liu B.S., Hu G., Wang S.G., Desneux N. (2024). Regulation of three novel pepper thiothiazolidinones on the fecundity of *Spodoptera frugiperda*. Pestic. Biochem. Phys..

[36] Tang B., Yang M.M., Shen Q.D., Xu Y.X., Wang H.J., Wang S.G. (2017). Suppressing the activity of trehalase with validamycin disrupts the trehalose and chitin biosynthesis pathways in the rice brown planthopper, *Nilaparvata lugens*. Pestic. Biochem. Phys..

[37] Yu H.Z., Huang Y.L., Lu Z.J., Zhang Q., Su H.N., Du Y.M., Yi L., Zhong B.L., Chen C.X. (2021). Inhibition of trehalase affects the trehalose and chitin metabolism pathways in *Diaphorina citri* (Hemiptera: Psyllidae). Insect Sci..

[38] Tatun N., Singtripop T., Sakurai S. (2008). Dual control of midgut trehalase activity by 20-hydroxyecdysone and an inhibitory factor in the bamboo borer *Omhisa fuscidentalis* Hampson. J. Insect Phys..

[39] Leyva A., Quintana A., Sánchez M., Rodríguez E., Cremata J., Sánchez J. (2008). Rapid and sensitive anthrone-sulfuric acid assay in microplate format to quantify carbohydrate in biopharmaceutical products: Method development and validation. Biol. J. Int. Assoc. Biol. Standard..

[40] Tatun N., Singtripop T., Tungjitwitayakul J., Sakurai S. (2008). Regulation of soluble and membrane-bound trehalase activity and expression of the enzyme in the larval midgut of the bamboo borer *Omphisa fuscidentalis*. Insect Biochem. Insect Mol. Biol..

[41] Zhao S.Y., Yang X.M., He W., Zhang H.W., Jiang Y.Y., Wu K.M. (2019). Ovarian Development Grading and Reproductive Potential Prediction Method for *Spodoptera frugiperda*. Plant Protect..

[42] Xu H.H., Wang J.L., Wei J.Q., Zhu J., Lin F. (2020). Genetic regulation and reproductive characteristics interference of insect populations and their application prospects in the prevention and control of fall armyworms in grasslands. J. South China Agr. Univ..

[43] Zheng H.Y., Fan S.F. (2022). Research progress on the function and mechanism of action of insect lipid hormones. Chin. J. Biol. Control Prev..

[44] Liu Y., Yang F., Wan S., Wang X., Guan L., Li Y., Xu C., Xie B., Wang S., Tan X. (2025). Comparative transcriptomic and metabolomics analysis of ovary in Nilaparvata lugens after trehalase inhibition. BMC Genom..

[45] Yamada T., Habara O., Yoshii Y., Matsushita R., Kubo H., Nojima Y., Nishimura T. (2019). The role of glycogen in development and adult fitness in Drosophila. Development.

[46] Tatun N., Tungjitwitayakul J., Sakurai S. (2016). Developmental and lethal effects of trehalase inhibitor (validamycin) on the *Tribolium castaneum* (Coleoptera: Tenebrionidae). Ann. Entomol. Soc. Am..

[47] Tang B., Wei P., Zhao L., Shi Z., Shen Q., Yang M., Xie G., Wang S. (2016). Knockdown of five trehalase genes using RNA interference regulates the gene expression of the chitin biosynthesis pathway in *Tribolium castaneum*. BMC Biotechnol..

[48] Zhu B.Q., Du X. (2019). Research progress on insect trehalose and its inhibitors. Biochemistry.

[49] Liu X.Y., Wang S.S., Zhong F., Zhou M., Jiang X.Y., Cheng Y.S., Dan Y.H., Hu G., Li C., Tang B. (2022). Chitinase (CHI) of *Spodoptera frugiperda* affects molting development by regulating the metabolism of chitin and trehalose. Front. Physiol..

[50] Siriwattanarungsee S., Sukontason K.L., Olson J.K., Chailapakul O., Sukontason K. (2008). Efficacy of neem extract against the blowfly and housefly. Parasitol. Res..

[51] Li W., Wu Y.R., Wang X.M., Chen Z.L., Liu J., Zhao Y., Peng Y., Zhu Y. (2024). Transgenerational effect of a tea saponin-matrine mixture promotes fecundity and alters associated bacteria in the fall armyworm *Spodoptera frugiperda*. Ind. Crop Prod..

[52] Souza-Ferreira P.S., Mansur J.F., Berni M., Moreira M.F., Santos R.E., Araújo H.M.M., Souza W., Ramos I.B., Masuda H. (2014). Chitin deposition on the embryonic cuticle of *Rhodnius prolixus*: The reduction of CHS transcripts by CHS-dsRNA injection in females affects chitin deposition and eclosion of the first instar nymph. Insect Biochem. Mol. Biol..

[53] Mansur J.F., Alvarenga E.S., Figueira-Mansur J., Franco T.A., Ramos I.B., Masuda H., Melo A.C.A., Moreira M.F. (2014). Effects of chitin synthase double-stranded RNA on molting and oogenesis in the Chagas disease vector *Rhodnius prolixus*. Insect Biochem. Mol. Biol..

[54] Catchot B., Anderson C.J., Gore J., Jackson R., Rakshit K., Musser F., Krishnan N. (2020). Novaluron prevents oogenesis and oviposition by inducing ultrastructural changes in ovarian tissue of young adult *Lygus lineolaris*. Pest Manag. Sci..

[55] Harðardóttir H.M., Male R., Nilsen F., Dalvin S. (2021). Chitin synthases are critical for reproduction, molting, and digestion in the salmon Louse (*Lepeophtheirus salmonis*). Life.

[56] Santos R., Alves-Bezerra M., Rosas-Oliveira R., Majerowicz D., Meyer-Fernandes J.R., Gondim K.C., Meyer-Fernandes J.R., Gondim K.C. (2012). Gene identification and enzymatic properties of a membrane-bound trehalase from the ovary of *Rhodnius prolixus*. Arch. Insect Biochem. Physiol..

[57] Shimada S., Yamashita O. (1979). Trehalose absorption related with trehalase in developing ovaries of the silkworm, *Bombyx mori*. J. Comp. Physiol. B-Biochem. Syst. Environ. Phys..

[58] Yu H.Z., Zhang Q., Lu Z.J., Deng M.J. (2022). Validamycin treatment significantly inhibits the glycometabolism and chitin synthesis in the common cutworm, *Spodoptera litura*. Insect Sci..

[59] Adhav A.S., Kokane S.R., Joshi R.S. (2018). Functional characterization of *Helicoverpa armigera* trehalase and investigation of physiological effects caused due to its inhibition by validamycin A formulation. Int. J. Biol. Macromol..

[60] Tan Y.A., Zhao X.D., Sun H.J., Zhao J., Xiao L.B., Hao D.J., Jiang Y.P. (2021). Phospholipase C gamma (PLCγ) regulates soluble trehalase in the 20E-induced fecundity of *Apolygus lucorum*. Insect Sci..

[61] Lin Y., Meng Y., Wang Y.X., Luo J., Katsuma S., Yang C.W., Banno Y., Kusakabe T., Shimada T., Xia Q.Y. (2013). Vitellogenin receptor mutation leads to the oogenesis mutant phenotype "scanty vitellin" of the silkworm, *Bombyx mori*. J. Biol. Chem..

[62] Sun Y., Xiao L., Cao G., Zhang Y., Xiao Y., Xu G., Zhao J., Tan Y., Bai L. (2016). Molecular characterisation of the vitellogenin gene (*AlVg*) and its expression after *Apolygus lucorum* had fed on different hosts. Pest Manag. Sci..

[63] Tufail M., Takeda M. (2008). Molecular characteristics of insect vitellogenins. J. Insect Phys..

[64] Kono Y., Takahashi M., Matsushita K., Nishina M., Kameda Y. (2001). Inhibition of oocyte development by a trehalase inhibitor, validoxylamine A, in *Periplaneta americana*. Med. Entomol. Zool..

[65] Qin Q., Zhang B., Fang B., Chang Y., Li X., An S., Zhao W. (2025). Juvenile hormone controls trehalose metabolism by regulating trehalase 2 activity in ovarian development of *Helicoverpa armigera*. Insect Mol. Biol..

[66] Chen J., Tang B., Chen H., Yao Q., Huang X., Chen J., Zhang D., Zhang W. (2010). Different functions of the insect soluble and membrane-bound trehalase genes in chitin biosynthesis revealed by RNA interference. PLoS ONE.

[67] Jiang X.Y., Zhong F., Chen Y., Shi D.M., Chao L., Yu L.H., He B., Xu C.D., Wu Y., Tang B. (2023). Novel compounds ZK-PI-5 and ZK-PI-9 regulate the reproduction of *Spodoptera frugiperda* (Lepidoptera: Noctuidae), with insecticide potential. J. Econ. Entomol..

